# Cytostatic and Antiproliferative Activities of F5 Fraction of* Crinum amabile* Leaf Chloroform Extract Showed Its Potential as Cancer Chemotherapeutic Agent

**DOI:** 10.1155/2019/7521504

**Published:** 2019-04-11

**Authors:** Chung Pin Lim, Mun Fei Yam, Mohd. Zaini Asmawi, Voon Kin Chin, Nurul Hayah Khairuddin, Yoke Keong Yong, Haniza Hassan, Rusliza Basir

**Affiliations:** ^1^Faculty of Medicine and Health Sciences, Universiti Putra Malaysia, 43400 Serdang, Selangor Darul Ehsan, Malaysia; ^2^School of Pharmaceutical Sciences, Universiti Sains Malaysia, 11800 Minden, Pulau Pinang, Malaysia; ^3^School of Biosciences, Faculty of Health and Medical Sciences, Taylor's University Lakeside Campus, 47500 Subang Jaya, Malaysia; ^4^Faculty of Veterinary Medicine, Universiti Putra Malaysia, 43400 Serdang, Selangor Darul Ehsan, Malaysia

## Abstract

Medicinal plants have been considered as promising sources of drugs in treating various cancers.* Crinum amabile (C. amabile)*, a plant species from the Amaryllidaceae family, is claimed to be a potential source for cancer chemotherapeutic compounds. Here, we aimed to investigate the potential of* C. amabile* as an anticancer agent. Dried leaves of* C. amabile* were serially extracted and our findings showed that chloroform extract (CE) was shown to exhibit cytotoxic effect against all cancer cell lines used. This active extract was further fractionated in which F5 fraction was shown to possess the highest cytotoxicity among all fractions. F5 fraction was then tested in-depth through Annexin V/FITC apoptosis and DNA fragmentation assays to determine its apoptotic effect on MCF-7 cells. Results revealed that F5 fraction only showed induction of cell apoptosis starting at 72-hour treatment while DNA fragmentation was not detected at any of the concentrations and treatment periods tested. Meanwhile, cell proliferation assay revealed that F5 fraction was able to inhibit normal cell proliferation as well as VEGF-induced cell proliferation of normal endothelial cell (HUVECs). In conclusion, F5 fraction from* C. amabile* leaf CE was able to exhibit cytostatic effect through antiproliferation activity rather than induction of cell apoptosis and therefore has the potential to be further investigated as an anticancer agent.

## 1. Introduction

Cancer remains as the leading cause of death worldwide, regardless of regions and socioeconomic levels. The incidence of cancer is presumed to escalate owing to age, population growth, and adoption of an unhealthy lifestyle. According to WHO, more than 8.8 million deaths were recorded in 2015, with nearly 70% of them being from low- and middle-income countries [1]. Considerable efforts have been allocated to search for new drugs for the treatment and prevention of cancers each year. Nevertheless, the recurrence of tumor cells and the adverse effects of chemotherapy drugs have hindered the efficacy of cancer drugs treatment [2]. Hence, there is a constant need to actively search for an alternative in anticancer therapies such as viral-mediated targeted gene therapy [3].

From the dawn of ancient medicine, medicinal plants, as well as traditional remedies, have been served as potential sources of bioactive compounds to treat many illnesses [4]. The beneficial medicinal effects typically result from the combinations of secondary products present in the medicinal plants, which are mostly alkaloids, steroids, tannins, phenol compounds, flavonoids, steroids, resins, and fatty acids [5]. Of all, alkaloid has received the most attention since it is highly bioactive [6].

Programmed cell death (PCD) is the crucial process which governs cell homeostasis, cell development, and cell defense mechanisms [7, 8]. PCDs can be divided into three distinct types: cell apoptosis, cell necrosis, and cell autophagy [8]. Among them, cell apoptosis is the most extensively studied. According to Kerr et al. (1972), apoptosis is an important endogenous cellular regulator [9] in maintaining homeostasis of the human body and responsible for the elimination of damaged, unhealthy, and senescent cells from the body [10, 11] without causing tissue damage and necrosis-associated inflammation [12]. Briefly, cell apoptosis is characterized by a series of morphological and biochemical changes in the cells which ultimately caused the demise of the cell [13]. These morphological changes include membrane blebbing; change in mitochondrial membrane potential and the resulting loss of mitochondrial integrity; cytochrome c translocation; cell membrane phospholipid asymmetry (but not increased cell permeability); dilation of endoplasmic reticulum; condensation of cytoplasm and cell shrinkage; chromatin condensation; internucleosomal DNA cleavage and cell fragmentation; loss of cell membrane permeability; and finally the emergence of apoptotic bodies [9, 14–18]. The entire cell apoptosis process may occur from several hours to days, depending on the strength of stimuli and the types of cells involved [19].

*C. amabile*, or better known by local as Purple Giant Spider-Lily or swamp lily, is a type of terrestrial herbaceous classified under plant family: Amaryllidaceae [20]. It was claimed to be a potential source of beneficial chemotherapeutic compounds [21–25]. A study has shown that alkaloids isolated from* C. amabile* possess a myriad of pharmacological and biological activities [26]. For example, lycorine was shown to suppress tumor cell growth and reduce cell survival via cell cycle arrest and induction of apoptosis [27, 28]. While most of the studies were focusing on the alkaloids isolated from its bulbs [5, 25, 29], no evidence was found to describe the anticancer effects of its leaves. Therefore, the current research was undertaken to determine the cytotoxic effects of various extracts and fractions of* C. amabile* leaves on various cancer cell lines and to investigate whether they kill the cells through induction of cell apoptosis or by inhibition of cell proliferation through antiangiogenesis pathway.

## 2. Materials and Methods

### 2.1. Cell Lines and Cultures

Five different human cancer cell lines, namely, MCF-7 (breast carcinoma with estrogen receptor (ER+)), MDA-MB-231 (breast carcinoma without estrogen receptor (ER-)), HT-29 (colon adenocarcinoma), HCT-16 (colon carcinoma), and Reh (acute lymphoma leukemia), were employed in this study. MCF-7, HT-29, and HCT-116 cells were kind gifts by Dr. Amin Malik Shah Bin Abdul Majid (Universiti Sains Malaysia), while MDA-MB-213 and Reh cells were purchased from American Type Culture Collection (ATCC, USA). MCF-7 cells were cultured in Dulbecco's Modified Eagle's Medium (DMEM) complete medium (Gibco, USA) while MDA-MB-231 cells were cultured in Leibovitz's L-15 complete medium (Gibco, USA). HT-29, HCT-116, and Reh cells were cultured using RPMI complete medium (Gibco, USA). All complete media were supplemented with 10% heat-inactivated fetal bovine serum (FBS) (Gibco, USA) and 100U/mL Penicillin-Streptomycin (Gibco, USA). All the cells were incubated at 37°C with a humidified atmosphere containing 5% of CO_2_, except for MDA-MB-231 cells which were cultured without CO_2_. All the cells were subcultured every 2-3 days to reach 90% confluence.

### 2.2. Collection of Leaves Sample

The fresh leaves of* C. amabile* were collected, cleaned, and dried before grounded into powder. The weight of the leaf powder was measured. One leaf and flower sample were collected and a voucher specimen was deposited under the number SK 3365/18 at Biodiversity Unit, Institute of Bioscience, Universiti Putra Malaysia (UPM).

### 2.3. Serial Extraction Using Soxhlet's Apparatus

The leaf powder of* C. amabile *was extracted in a series of reflux with different organic solvents, starting from nonpolar solvent to polar solvent, namely, Petroleum Ether (Fisher Scientific, Loughborough, UK), Chloroform (Fisher Scientific, Loughborough, UK), Methanol (R&M Chemicals, Essex, UK), and finally water. After the first extraction, petroleum ether extract (PE) was filtered and concentrated. The marc was dried and prepared for subsequent extraction. All the extracts were frozen at -80°C for 48 hours and then evaporated to dryness using freeze-dryer for another 48 hours. Lastly, each extract was weighed and each yield was calculated. All four extracts were stored in a desiccator [30] and the stock solution (20 mg/mL) for each extract was prepared in 100% dimethyl sulfoxide (DMSO) (Sigma-Aldrich, USA). All stock solutions were kept in -20°C prior to use in airtight containers to prevent microbial growth and deterioration caused by moisture [31].

### 2.4. Fractionation Using Column Chromatography

Ten grams of chloroform extract (CE) was dissolved in pure chloroform (Fisher Scientific, Loughborough, UK) together with 20 g of silica gel (Merck, Germany). The solution was mixed well and evaporated. The powdered CE obtained was then placed on top of the packed silica column. A series of different ratio of petroleum ether, chloroform, and methanol mixtures (Fisher Scientific, Loughborough, UK) as depicted in Table 1 were prepared. The silica column was subsequently eluted with this series of mixtures in increased polarity. Each successive eluate was collected and concentrated.

### 2.5. Thin Layer Chromatography (TLC) Profiling

TLC profiling was performed right after column chromatography fractioning using TLC plate (Merck, Germany) [32]. Briefly, TLC plates of 5x10cm were prepared. A line with 1cm from the bottom edge of the plate was drawn where spots of concentrated eluates were seeded. Different mobile phases were then prepared like Petroleum Ether-Ethyl Acetate (4:1, 3:2, 1:1 and 1:2), Ethyl Acetate-Methanol (1:1), and Chloroform-Methanol (3:2, 1:1 and 2:3) and were poured into a sealed TLC chamber. The chamber was left to equilibrate. Next, the spotted TLC plates were placed vertically into the solvent vapor-saturated chamber. The process was terminated right before the mobile solvent reached the top edge of the plate. TLC plates were then visualized under long (365nm) and short (254nm) wavelength using a UV transilluminator. The R_f_ value for each separated spot was calculated using the following formula:

(1) $$\begin{array}{c} R_{f}=\frac{distance\;\;traveled\;\;by\;\;the\;\;compound}{distance\;\;traveled\;\;by\;\;the\;\;mobile\;\;phase} \end{array}$$

Those eluates with more than 85% similarities in TLC profiles were pooled together as one fraction. The stock solution of 20 mg/mL for each fraction was prepared in 100% DMSO and kept in -20°C prior to use.

### 2.6. Cell Cytotoxicity Assay

Cell cytotoxicity was assessed through CellTiter 96® Aqueous Non-Radioactive Cell Proliferation Assay (MTS Assay) (Promega, USA) as per manufacturer's instructions [33]. Cell numbers were adjusted after cell counts (MCF-7: 8.0x10^3^ cells/well; HT-29: 1.0x10^4^ cells/well; HCT-116: 2.0x10^4^ cells/well; MDA-MB-231 and Reh: 2.5x10^4^ cells/well) using their respective complete medium without phenol red. 90*μ*L of cell solutions were inoculated into a 96-well plate and incubated overnight. After incubation, 10*μ*L of different concentrations of various extracts and fractions were introduced into respective well. 5-fluorouracil (5-FU) (donated by Penang General Hospital, Malaysia) was used as reference drug for comparison. After 48 hours, 20*μ*L of MTS reagents was added into each well followed by incubation for another 2-5 hours (HT-29: 2 hours; HCT-116: 3 hours; MCF-7, MDA-MB-231, and Reh: 5 hours). The absorbance value for each well was measured at 490nm and 630nm (reference wavelength) using a microplate reader. The percentage of inhibition for each extract and fraction was calculated as follows:

(2) $$\begin{array}{c} Percentage\;\;of\;\;inhibition\;\left(\;\%\;\right)=\left[\;\frac{\left(A_{n}-A_{s}\right)}{A_{n}}\;\right]\times 100\% \end{array}$$

whereby

A_n_ = absorbance of negative control

A_s_ = absorbance of test samples (extracts, fractions, or 5-FU)

### 2.7. Annexin V/PI Dual Staining Assay

Cell apoptosis was examined using a commercially available Annexin V/PI dual staining assay (Biovision Research Products, USA). Briefly, MCF-7 cells were cultured (7.1 x 10^5^ cells) overnight in 5mL complete medium. The cells were treated with F5 fraction at IC_50_ and double IC_50_ for 12, 24, 48, 72, and 96 hours. After treatment, all the cells were washed twice with 5mL cold PBS and centrifuged at 1.3 x 10^4^ rpm for 15 minutes. The cell numbers for each treatment were adjusted to 2.0 x 10^5^ cells/mL in which 1mL of each cell solution was resuspended in 0.5mL binding buffer. 5*μ*L of Annexin V and PI was added followed by incubation for 5 minutes at room temperature in the dark. Lastly, the stained cells were kept in an ice box and immediately analyzed by the flow cytometer (Becton Dickinson FACS Canto II Flow Cytometer, BD Biosciences). At least 1.0 x 10^4^ events per tube were evaluated using a FACS Canto II Flow Cytometer with Annexin V fluorescence (emission 520nm) detected at FL1 and FL4 channels while PI fluorescence (emission 550nm) is detected at FL2 and FL3 channels. One percent of DMSO was used as negative control while 5-FU and tamoxifen (Sigma-Aldrich, USA) were used as positive controls in this study. The study was repeated in three independent experiments. All results were analyzed using the BD FACS Diva software (Cell Quest). The morphological changes of treated MCF-7 cells were observed under the light microscope at 100x magnifications.

### 2.8. DNA Fragmentation Assay

DNA fragmentation assay was adapted from Sellins and Cohen, (1987) with slight modification [34]. The MCF-7 cells were cultured overnight and treated with F5 fraction at IC_50_ and double IC_50_ for 24, 48, 72, 96, and 120 hours. The cells were harvested and centrifuged at 1 x 10^3^ rpm for 3 minutes. The cell pellets were resuspended in 467 *μ*L Tris-EDTA (TE) buffer (10 mM Tris-HCl, 1 mM EDTA, pH 8.0) (Sigma-Aldrich, USA) and treated with 30 *μ*L of 10% SDS solution (Sigma-Aldrich, USA) and 3 *μ*L of 20 mg/mL proteinase K (Sigma-Aldrich, USA) an hour at 40°C. After incubation, each cell solution was resuspended in phenol-chloroform-isoamyl alcohol mixture and centrifuged at 1.3 x 10^4^ rpm for 5 minutes. Then, the upper layer of each cell solution was collected and an equal amount of saturated trichloromethane (Sigma-Aldrich, USA) was added. The solutions were mixed well and centrifuged at 1.3 x 10^4^ rpm for another 5 minutes. Again, the upper layer of each cell solution was collected and 0.1 volume of sodium acetate (QREC, Malaysia) and 1mL of 100% ethanol (R & M Chemicals, UK) were added. All cell solutions were mixed thoroughly and stored at -20°C overnight. Subsequently, the cell solutions were thawed and centrifuged at 1.3 x 10^4^ rpm for 20 minutes at 4°C. The DNA pellets obtained were washed with 1 mL of 70% cold ethanol and centrifuged at 1.3 x 10^4^ rpm for 15 minutes at 4°C. The washing process was repeated at least twice. Finally, the DNA pellets were air-dried and dissolved in 100*μ*L of TE buffer. DNA concentrations were determined as A_260_ x dilution factor x 50 *μ*g/mL, using an UV spectrophotometer while DNA purity was calculated based on A_260_/A_280_ ratio. Only DNA samples with purity higher than factor 1.7 were used. Here, all the DNA samples were resolved by electrophoresis on 1.8% ethidium bromide-stained agarose gel at 80V for 30 minutes and visualized under an UV transilluminator at 254nm wavelength. One percent of DMSO was served as negative control while 5-FU and tamoxifen were used as positive controls. The assay was repeated in three independent experiments. All the morphological changes of treated MCF-7 cells were observed under the light microscope at 100x magnifications.

### 2.9. Cell Proliferation Assay

The proliferation capacity of HUVEC cells was assessed according to Li et al., (2016) with slight modifications [35]. HUVEC cells were plated at 3 000 cells/well in 150 *μ*L into a 96-well plate and cultured for 24 hours. Next day, the old media were discarded and the cells were treated with 0.25 *μ*g/mL of F5 fraction, with and without stimulation of VEGF (20 ng/mL) using 0.5% FBS containing-media for 24, 48, and 72 hours, respectively. The treatment media were prepared fresh and renewed daily. Finally, 30 *μ*L of MTT solution was added to each well and incubated for another four hours prior to absorbance reading being measured at wavelength of 570 nm. Each concentration was performed in triplicates and the full-set experiment was repeated for at least three times. Absorbance readings were normalized to the levels in the negative control (DMSO) to determine the percentage of viable cells [36]. Here, the percentage of DMSO used in the experiment was 0.01%. The results obtained were compared between VEGF treated and non-VEGF treated.

### 2.10. Statistical Analysis

All results were expressed as mean ± standard deviation (SD) or standard error mean (SEM) whichever is appropriate. Statistical analyses were performed by using one-way analysis of variance (ANOVA), with post hoc Dunnett's test using the Statistical Package for Social Science Version 22 software (SPSS Inc., Chicago, IL). P values of less than 0.05 (P < 0.05) were considered as significant in all cases.

## 3. Results

### 3.1. Extraction Yields

The weights of extracts obtained were 155.2908g, 84.2454g, 443.2597g, and 72.2390g for petroleum ether, chloroform, methanol, and water extract, respectively. The yields calculated were 7.76%, 4.21%, 22.16%, and 6.02% for the same extracts. Meanwhile, the weights for the six fractions obtained were 1.4654g, 1.1029g, 1.2173g, 0.7702g, 1.9706g, and 1.0416g for F1 to F6 fraction, respectively. The yields calculated were 14.65%, 11.03%, 12.17%, 7.70%, 19.71%, and 10.42% for the respective fractions.

### 3.2. Cytotoxicity of* C. amabile* on Human Cancer Cells

**Figure 1:**
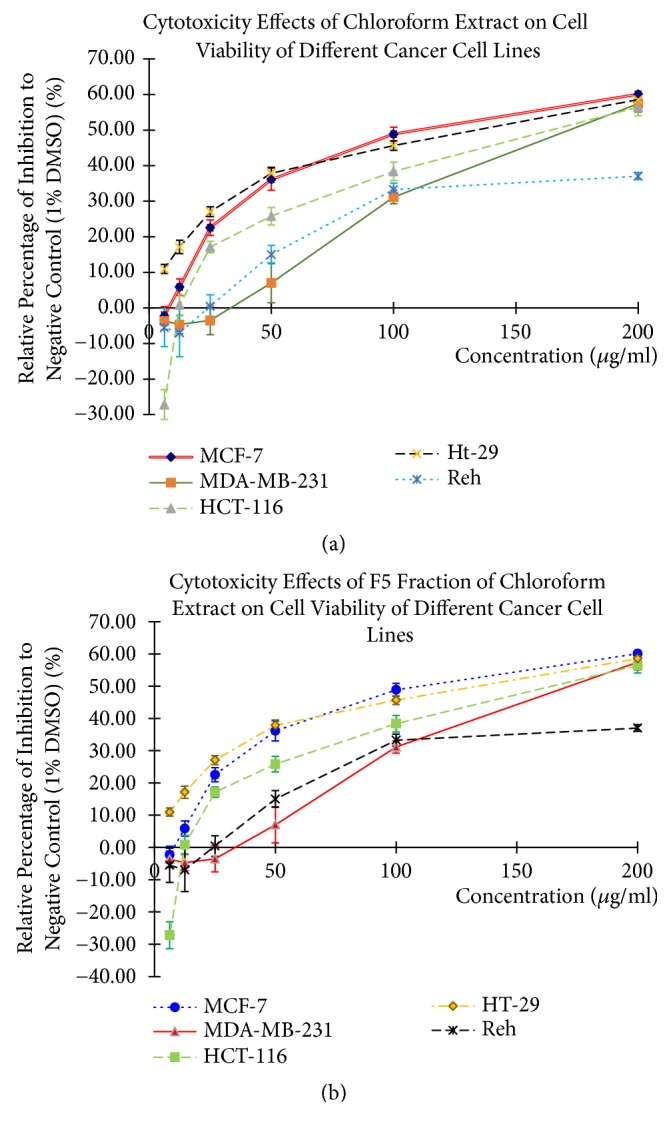
The cytotoxicity effects of (a) chloroform extract (CE) and (b) F5 fraction on various cell lines: MCF-7, MDA-MB-231, HCT-116, HT-29, and REH, assessed using MTS Assay. All extract and fraction were exposed for 48 hours at different concentration, ranged from 6.25 *μ*g/mL-200.00 *μ*g/mL. Each point represents an average of three individual experiments of triplicate samples (n=9).

The cytotoxicity of* C. amabile* leaf extracts and its fractions were screened on five different human cancer cell lines and the results were summarized in Table 2. Only chloroform extract (CE) showed concentration-dependent cytotoxicity effects on MCF-7, MDA-MB-231, HCT-116, and HT-29 (Figure 1(a)). However, CE was less effective against Reh cells, followed by petroleum extract (PE) and methanol extract (ME), which produced slight cytotoxicity, while water extract (WE) showed no effect to all the human cancer cells lines used even at the highest concentration tested 200 *μ*g/mL. Their IC_50_ values determined were higher than 200 *μ*g/mL. Meanwhile, F5 fraction exhibited the highest, pronounced cytotoxicity against all the human cancer cell lines (Figure 1(b)). Other fractions (F1, F2, F3, F4, and F6), on the other hand, produced low cytotoxic effects on MDA-MB-213 cell but with moderate cytotoxic effects on HCT-116, HT-29, and Reh cells (Figure 1(b)). Finally, 5-FU exhibited potent cytotoxicity effects on MCF-7, HCT-116, and Reh cells but less effective against MDA-MB-231 cells.

### 3.3. Cell Apoptosis by* C. amabile*

**Figure 2:**
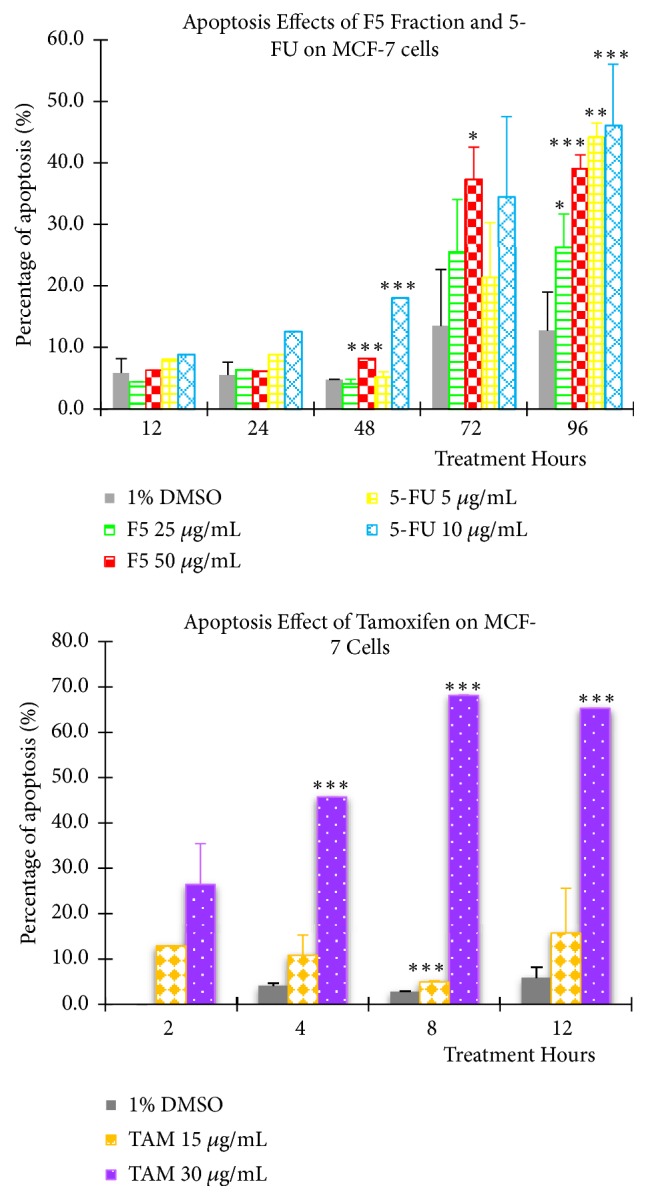
The percentages of apoptosis induced by (A) F5 fraction and 5-FU and (B) tamoxifen on MCF-7 cell line at IC_50_ and double IC_50_, respectively, using Annexin V-FITC and PI double staining kit. All samples (F5 Fraction, 5-FU and tamoxifen) were treated at different time point and each result was obtained from flow cytometry analysis. Significant level: *∗* * p*<0.05, *∗∗* * p*<0.01, and *∗∗∗* * p*<0.001.

**Figure 3:**
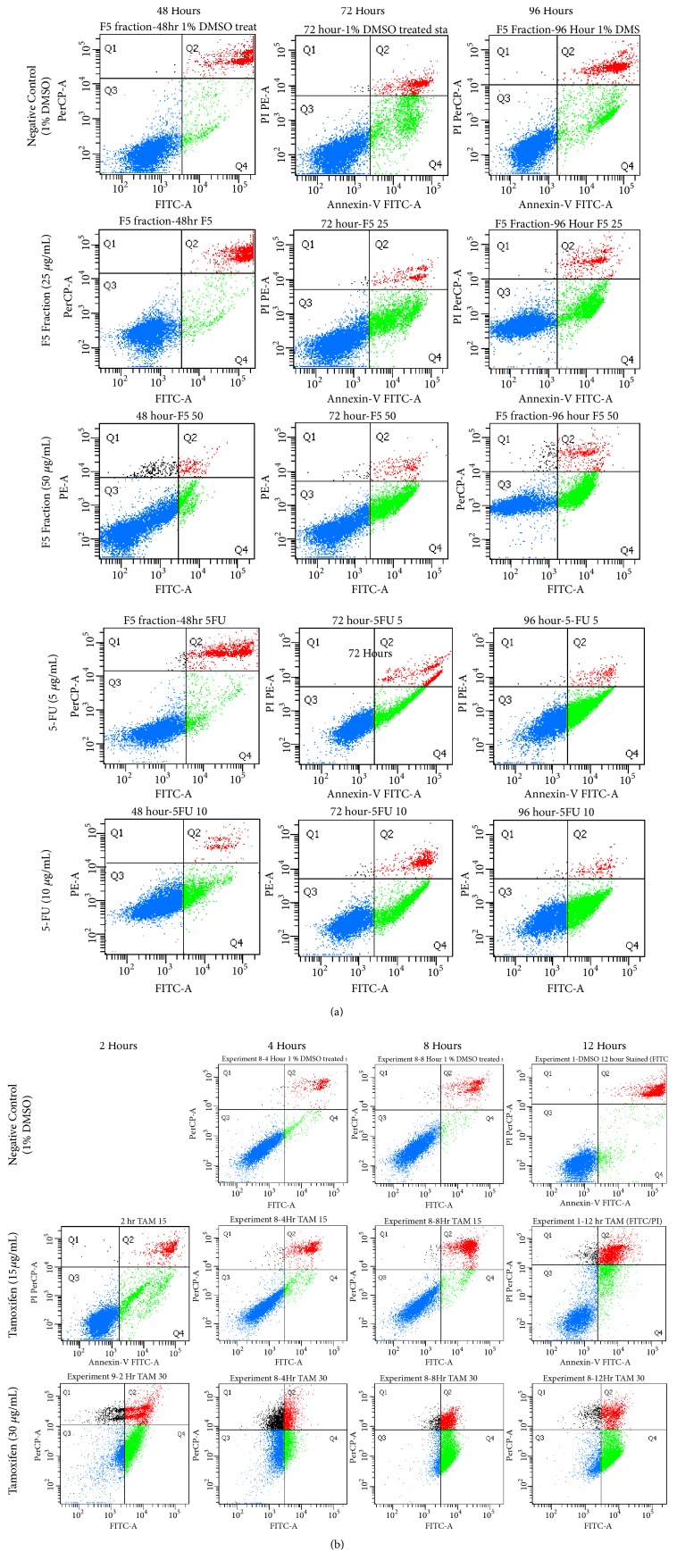
(a) Scatterplots obtained from Annexin V-FITC apoptosis assay for F5 Fraction and 5-FU, treated at IC_50_ and double IC_50_, at various time point (48, 72 and 96 hours) using FCM software. (b) Scatterplots obtained from Annexin V-FITC apoptosis assay for tamoxifen, treated at IC50 and double IC50, at various time point (48, 72 and 96 hours) using FCM software.

When the cells were treated with F5 fraction at IC_50_: 25 *μ*g/mL for 12 hours, no significant cell apoptosis (4.4%) was detected. Slowly, the percentage of cell apoptosis increased from 6.3% at 24 hours to 4.1±0.8% at 48 hours and substantially increased to 25.5±8.5% at 72 hours before reaching a high of 26.3±5.4% at the end of the 96^th^ hour treatment. During treatment with the double IC_50_ of 50 *μ*g/mL, a minimum cell apoptosis population was observed with percentage of 6.3% at 12 hours which later increased to 6.1% at 24 hours, 8.2% at 48 hours, 37.3±5.2% at 72 hours, and, finally, 39.0±2.3% at the 96^th^ hour of treatment. However, the increments were only statistically significant at 48, 72 and 96 hours of treatments for double IC_50_ while IC_50_ was only significant at 96-hour treatment compared to the negative control (Figures 2 and 3).

For comparison, the same experimental condition was repeated and applied to two positive controls, 5-FU and tamoxifen. At the 12^th^ hour of 5-FU treatment, the percentages of cell apoptosis detected were 8.1% at 5 *μ*g/mL and 8.8% at 10 *μ*g/mL, respectively. Subsequently, cell apoptosis increased to 8.8%, 5.3±0.8%, 21.3±9.0%, and 44.2±2.3% for the 5 *μ*g/mL concentration while cell apoptosis for the 10 *μ*g/mL concentration was measured at 12.6%, 18.0%, 34.5±13.1%, and 46.0±10.0% for each of the 24, 48, 72, and 96 hours of treatments. Again, the results were only statistically significant at 96 hours treatment for IC_50_ and at 48 and 96 hours treatments for double IC_50_ when compared to the negative control, respectively.

On the other hand, tamoxifen induced a far greater apoptosis effect to MCF-7 cells with proportions of cell apoptosis identified for 2, 4, 8, and 12 hours of treatments measured at 26.5%, 45.7%, 68.1%, and 65.3%, respectively, when treated at double IC_50_ (30 *μ*g/mL). The results were statistically significant at 4, 8, and 12 hours of treatment. However, cells treated with tamoxifen at IC_50_ did not show any significant increase in cell apoptosis, for all the treatment periods except at 12 hours treatment compared to negative control. Lastly, MCF-7 cells treated with 1% DMSO produced only 4-10% cell apoptosis for all the intervals tested.

### 3.4. Effects of* C. amabile* on DNA Fragmentation

**Figure 4:**
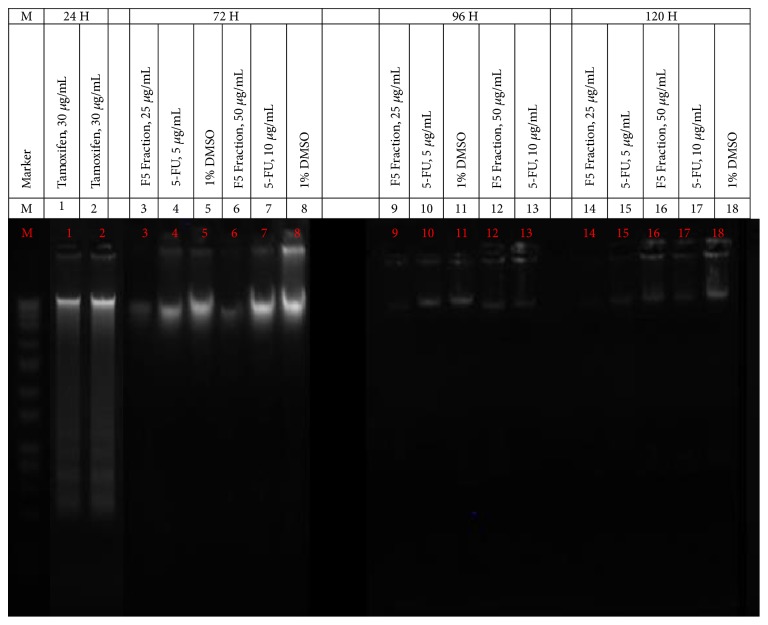
Gel electrophoresis showed DNA laddering formation of F5 fraction, 5-FU, and tamoxifen on MCF-7 cells, treated at IC_50_ and double IC_50_, for different treatment periods (24, 48, 72, 96, and 120 hours), during DNA fragmentation assay. Lane M: standard molecular size marker. At 24 hours of treatment, Lanes 1 and 2: tamoxifen, 30 *μ*g/mL. At 72 hours of treatment, Lane 3: F5 fraction, 25 *μ*g/mL, Lane 4: 5-FU, 5 *μ*g/mL, Lane 5: 1% DMSO, Lane 6: F5 Fraction, 50 *μ*g/mL, Lane 7: 5-FU, 10 *μ*g/mL, and Lane 8: 1% DMSO. At 96 hours of treatment, Lane 9: F5 fraction, 25 *μ*g/mL, Lane 10: 5-FU, 5 *μ*g/mL, Lane 11: 1% DMSO, Lane 12: F5 Fraction, 50 *μ*g/mL, and Lane 13: 5-FU, 10 *μ*g/mL. At 120 hours of treatment, Lane 14: F5 Fraction, 25 *μ*g/mL, Lane 15: 5-FU, 5 *μ*g/mL, Lane 16: F5 Fraction, 50 *μ*g/mL, Lane 17: 5-FU, 10 *μ*g/mL, and Lane 18: 1% DMSO.

From the results, no DNA fragmentation was evident on F5 fraction-treated MCF-7 cells at both concentrations in all of the intervals (Figure 4). Likewise, 5-FU-treated MCF-7 cells also failed to show any DNA ladder formation at both concentrations and intervals (Figure 4). All these treated cells including negative control produced intact genomes on agarose gel. Only MCF-7 cells treated with double IC_50_ of tamoxifen showed DNA ladder formation on agarose gel at the 24-hour treatment (Figure 4).

### 3.5. Effects of* C. amabile* on Morphological Features of MCF-7 Cells

**Figure 5:**
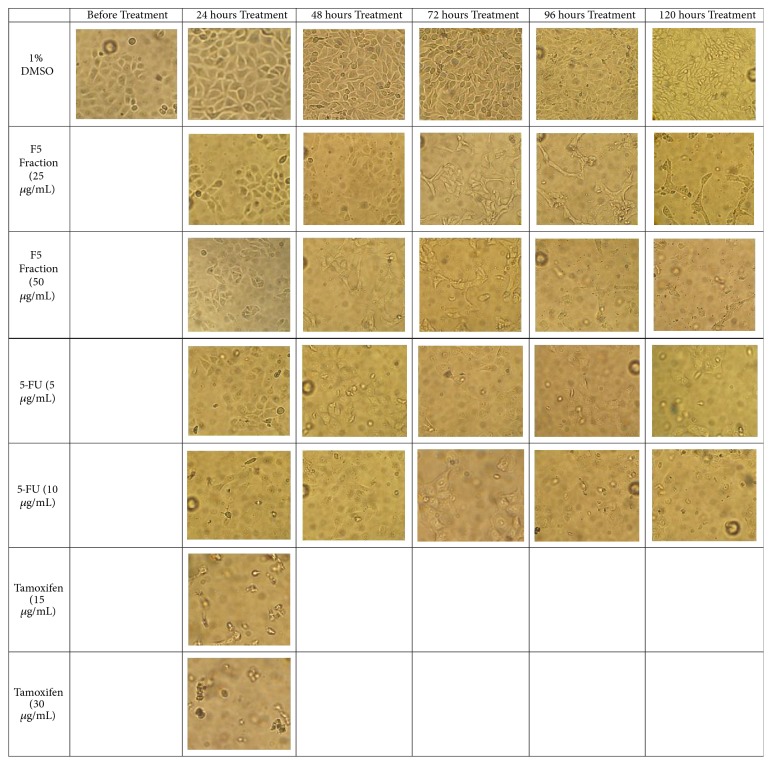
Effects of F5 fraction, 5-FU, tamoxifen, and negative control (1% DMSO) on MCF-7 cell morphology, treated at concentrations IC_50_ and double IC_50_ and respective incubation times, as observed under inverted light microscope at magnification 100x.

As depicted in Figure 5, the MCF-7 cells treated with F5 fraction at IC_50_ showed normal round and polygonal morphology at 24 and 48 hours of treatment. Subsequently after another day (72 hours), the cells started to show alteration on their cell morphology to become slightly elongated with a reduction in size. Later, most of the treated cells became elongated while other cells began to detach from the flask at 96-hour treatment. Finally, at 120-hour treatment, almost half of the cell population detached and formed round floating cells. Similar observation were recorded on MCF-7 cells treated at double IC_50_ but with sooner effect at 48 hours treatment and hence resulted in higher cell apoptosis at 72, 96, and 120 hours of treatments compared to negative control. A similar pattern was again observed in 5-FU treated cells. Instead of changing into elongated shape before cell detachment as observed in F5-fraction treated cells, the 5-FU treated cells turned semitransparent before being detached from the flask. Tamoxifen treated MCF-7 cells, on the other hand, showed cell detachment from the flask surface forming floating cells at 24-hour treatment. Higher frequency (more cell debris) was observed at double IC_50_ treated (Figure 5).

Meanwhile, MCF-7 cells treated with 1% DMSO displayed rounded and polygonal shape at 24, 48, and 72 hours of treatments and changed to irregular short rod shape during 96 and 120 hours of treatments (Figure 5).

### 3.6. Cell Proliferation Assay

**Figure 6:**
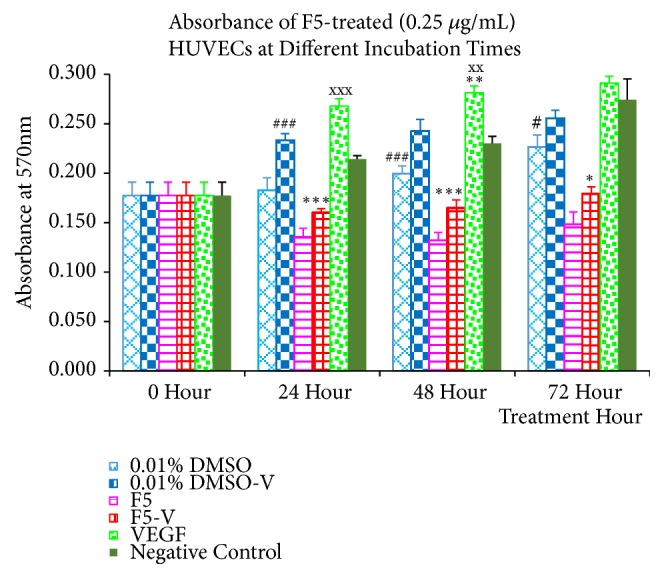
Antiproliferation effect (in terms of absorbance) of F5 fraction of* C. amabile* leaf CE on normal HUVECs through MTT assay. The HUVECs was treated with 0.25 *μ*g/mL of F5 fraction in 0.5% FBS media at various incubation periods (24, 48, and 72 hours). Each result represented an average of three independent experiments of triplicates (total n = 9), and plotted as mean ± SEM. Significance level was defined as follows: level 1* p*<0.05, level 2* p*<0.01, and level 3* p*<0.001, as compared against 0.01% DV (*∗∗∗*); F5 fraction (###); and normal control (^XXX^), respectively.

From Figure 6, normal control (0.5% FBS media) and VEGF-treated HUVECs showed greater elevation of absorbance readings as their incubation period increased. VEGF treatment demonstrated increase in absorbance readings from a baseline of 0.177 to 0.268 at 24 hours (151.42%) to 0.281 at 48 hours (158.98%) and finally to 0.291 at 72 hours (164.47%) of treatment while normal control displayed increment of absorbance signals from baseline to 0.214 at 24 hours (121.12%) to 0.230 at 48 hours (130.02%) and finally to 0.274 at 72 hours (154.94%).

Treatment groups using 0.01% DMSO (0.01% D) and 0.01% DMSO added with VEGF (0.01% DV) also demonstrated a rise in absorbance readings from 102.98% to 127.83% for 0.01% D and from 131.84% to 144.27% for 0.01% DV during the 24 to 72-hour treatment periods. Meanwhile, F5 fraction (F5) and F5-fraction with VEGF- (F5-V-) treated HUVECs experienced a decrease in absorbance signals to 0.135 (F5) and 0.160 (F5-V) at 24-hour treatment, followed by an increase to 0.148 (F5) and 0.179 (F5-V) at 72-hour treatment. Interestingly, the absorbance readings of cotreatment groups with VEGF (F5-V, 0.01% DV, and VEGF) were always higher than their respective counterparts (F5, 0.01% D, and normal control) at all test intervals.

## 4. Discussion

*Crinum *species have been used for medicinal purposes in several countries due to their alkaloidal constituents [22]. These alkaloids are usually referred to as ‘Amaryllidaceae alkaloids' because they are only isolated from this plant family [25]. Amaryllidaceae alkaloids exhibit a wide array of biological activities on the nervous system, cancers, and infectious diseases [20, 22, 29, 37–39]. This suggested that* crinum* species can be a potential candidate for the development of anticancer agents.

Cytotoxicity assay revealed that CE of* C. amabile* leaf and its F5 fraction were regarded as the most active extract and fraction against all human cancer cell lines tested with MCF-7 cells being the most susceptible. Similar result was obtained by Kumar Singh and Patra, 2018, where the CE of rhizomes of plant* Polygonatum verticillatum* (L.) All. (Ruscaceae) was shown to exhibit anticancer effect [31]. Here, the active cytotoxic compound present was suspected to be alkaloids since they are usually being extracted by nonpolar solvent such as chloroform [40] while other compounds such as phenolic, flavonoid, or tannin are not primarily associated with cytotoxic effect [31]. The active F5 fraction was able to inhibit the proliferation of MDA-MB-231 cells. These findings suggested that the cytotoxicity of F5 fraction was not cell-specific; it can inhibit both breast cancers regardless of their ER status. This offers an advantage over 5-FU. According to National Cancer Institute (NCI) standard, compounds which produce IC_50_ value lower than 20 *μ*g/mL are considered as active cytotoxic agents [41, 42]. According to definition, cytotoxic compounds are compounds that kill the cells directly while cytostatic compounds are substances that suppress cell-proliferation without killing the cells [43]. Since the IC_50_ value of F5 fraction was slightly above the NCI standard, F5 fraction was hypothesized to exhibit cytostatic activities rather than cytotoxic effects. And because of that, we extended our study to investigate whether F5 fraction inhibits MCF-7 cells through induction of cell apoptosis.

Annexin V/PI dual staining assay was employed to investigate whether the cytotoxic effect of F5 fraction on MCF-7 cells was mediated through cell apoptosis. Application of this double staining permits the differentiation between (1) viable cell with no Annexin V-FITC and PI binding (no fluorescence); (2) early apoptotic cell which has PS asymmetry stained by Annexin V-FITC, but not PI (emit only green fluorescence); and (3) late apoptosis and dead cell which has lost their plasma membrane integrity and take up both Annexin V-FITC and PI dye (emit green and red fluorescence) [44, 45]. From the results, there was a prominent increase in the population of early apoptosis at 72 hours treatment for F5 fraction at IC_50_. Additionally, double IC_50_ of F5 fraction showed a similar pattern but with higher effect, suggesting that the induction of cell apoptosis by F5 fraction in MCF-7 cells is concentration- and time-dependent. 5-FU treated MCF-7 cells also showed a similar pattern as F5 fraction but with higher effect, indicating that 5-FU is a stronger apoptosis inducer against MCF-7 cells compared to F5 fraction. Since the apoptosis induction pattern by F5 fraction was comparable to that of 5-FU, we speculated that F5 fraction may eventually induce cell apoptosis through a similar mechanism as the commercial drug 5-FU. Furthermore, 5-FU has been considered as a cytostatic agent [46] and thus strengthens our findings that F5 fraction may behave like a cytostatic drug. To confirm our hypothesis, another cytotoxic drug tamoxifen was tested. Tamoxifen is a nonsteroidal antiestrogen drug widely used in treatment of ER+ breast cancer [47]. It was shown to kill MCF-7 cells through induction of cell apoptosis [47]. Remarkably, tamoxifen was able to induce cell apoptosis at 12 hours of treatment at IC_50_ and as early as 2 hours at double IC_50_. The apoptotic cells produced were equivalent to that obtained for F5 fraction at 96-hour treatment. Comparison between tamoxifen and F5 fraction indicated that F5 fraction was not considered to be an apoptosis inducer of MCF-7 cells or at least not in the true sense. A possible explanation is that F5 fraction may not kill the cells through direct induction of apoptosis but instead inhibited their proliferation until a point where the cells could no longer sustain themselves further and die slowly through cell apoptosis after 48 hours. This hypothesis explain why the percentage of cell apoptosis caused by F5 fraction only started to increase at 72-hour treatment and justified its cytostatic result from the previous MTS cytotoxicity assay. Therefore, another apoptosis DNA fragmentation assay was included in the study.

DNA fragmentation is considered as the late event in the progression of cell apoptosis [48]. During DNA fragmentation, the cell chromatins are cleaved by the activated endonuclease enzymes into small DNA fragments of approximately 180 bp and their multiples in length [9, 49, 50]. This feature can then be detected using gel electrophoresis in DNA fragmentation assay [49]. This assay uses electric current to migrate the negatively charged DNA fragments through the agarose gel matrix from the negative end (anode) to the positive end (cathode) and hence separate the different sizes of DNA fragments whereby the larger fragments will move slower while the small fragments move faster and are deposited at the bottom end to form a pattern of DNA ladder [49, 51]. From this, DNA laddering pattern can be used as another definitive indication or a hallmark to detect cell apoptosis [49, 52, 53]. Otherwise, it may serve as a complementary assay to substantiate the result of other cell apoptosis assays as well as to differentiate between cell apoptosis and necrosis since during cell necrosis, the cell chromatins are randomly cleaved into various lengths forming a smearing pattern.

Our findings revealed that DNA fragmentation was not detected in both F5 fraction and 5-FU treated cells, regardless of concentrations and treatment periods. Tamoxifen on the other hand showed otherwise. These observations demonstrated that F5 fraction and 5-FU were unable to induce cell apoptosis on MCF-7 cells. These findings were in contrast with the results obtained from Annexin V-FITC apoptosis assay. Similar findings were reported by Marchal et al. (2004), in which the authors demonstrated that no DNA fragmentation was observed in breast cancer MCF-7 cells treated with 5-FU [54]. The authors further deduced that cell apoptosis by 5-FU is difficult especially on MCF-7 cells since it is one of the cells which DNA laddering is very difficult to be induced. They proved that only a few selective cytotoxic agents such as tamoxifen that can act preferentially through an apoptotic mechanism are able to induce DNA fragmentation in this cell [54]. Subsequently, several studies also verified that some cells undergo cell apoptosis without the presence of DNA fragmentation [55, 56]. In such cases, DNA fragmentation assay may not be suitable for being used in the detection of cell apoptosis by F5 fraction. Either testing on different cancer cells or further investigations using different assays is warranted to elucidate the actual anticancer mechanism lies behind the F5 fraction.

Under microscopic observation, F5 fraction treated MCF-7 cells demonstrated elongation of cell morphology beyond 72 hours of treatment and ended with detachment of cells to form floating cells at 120 hours of treatment, which were suspected to be apoptotic bodies. This observation illustrated that F5 fraction was able to inhibit the proliferation of MCF-7 cells and eventually caused cell apoptosis. 5-FU treated MCF-7 cells, on the other hand, showed enlargement and transparency of cell morphology rather than cell elongation seen in F5 fraction-treated cells. This observation verified that F5 fraction and 5-FU acted differently in their inhibition on MCF-7 cells, possibly through different inhibition pathways. Lastly, MCF-7 cells treated with the known cytotoxic drug, tamoxifen, showed rapid detachment of cells at both IC_50_ and double IC_50_, at 24-hour treatment. These observations were totally different from those of F5 fraction and 5-FU, proving that both F5 fraction and 5-FU were not true cytotoxic agents.

Proliferation and migration of endothelial cells are among the main and important events in angiogenesis process [35, 36]. Direct interference of these functions or endothelium cells themselves can serve as the first and most effective approach against angiogenesis. Since the induction of cell apoptosis pathway was being ruled out, thus, the present study was further designed to investigate the antiangiogenesis effect of F5 fraction on normal endothelial cell proliferation using the* in vitro* cell proliferation assay. In addition, its ability to antagonize the stimulatory action of VEGF on endothelial cell proliferation was also investigated [57, 58], since angiogenic factor-stimulated endothelial cell proliferation is one of the key components in angiogenic response [57].

From this experiment, VEGF-treated HUVECs shows induction of cell proliferation compared to normal control. However, the inductions were only significant at 24 (*P* > 0.001) and 48 hours (*P* < 0.001). The average absorbance reading for normal control during 72-hour treatment was comparable to that of VEGF-treated and hence no significance was detected. Meanwhile, the concentration of DMSO (0.01%) applied did not produce any significant inhibitory effect on the cell proliferation of HUVECs. This was proven from the result (Figure 6) where no significant difference was detected between treatment groups of 0.01% D and normal control at all the incubation periods tested. Comparison between the treatment groups of 0.01% D and F5-treated HUVECs showed significant differences at all the treatment hours tested (24 hours:* P* < 0.001; 48 hours:* P* < 0.001; 72 hours:* P* < 0.05). This result suggested that F5 fraction was able to stop the normal cell proliferation of HUVECs. On the other hand, comparison between treatment groups F5-V and F5-treated HUVECs showed no significance at all the treatment periods tested. In addition, the average absorbance values in F5-V treated HUVECs were consistent throughout all the incubation periods. This result pointed to the fact that VEGF was unable to promote cell proliferation to F5 fraction-treated HUVECs, or, in other words, F5 fraction was able to suppress the VEGF-induced cell proliferation of HUVECs. Identical results were observed by Angulo et al. (2011) and thereby proposed that the inhibitory action of F5 fraction is most likely due to specific blockade of VEGF-induced proliferation rather than being attributed to the inhibition of a common mitogenic signaling pathway [58]. Cumulative data from all these results demonstrated that F5 fraction (0.25 *μ*g/mL) was able to inhibit both normal cell proliferation and VEGF-induced cell proliferation of HUVECs.

## 5. Conclusions

F5 fraction of CE demonstrated the highest cytostatic activities against all the human cancer cell lines tested with MCF-7, ER+ breast cancer cells being the most susceptible. Additionally, F5 fraction also showed a similar inhibition effect on MDA-MB-231, ER- breast cancer cells, suggesting that the cytostatic effect of F5 fraction was non-cell-specific. This finding provided a noteworthy benefit in which novel promising cytotoxic compound which is effective against both ER+ and ER- breast cancer cells can be isolated from this plant. However, cell apoptosis of F5-treated cells was only detected at 72 hours of treatment using cell apoptosis Annexin V assay and no DNA laddering pattern was found in the DNA fragmentation assay, regardless of concentrations and treatment periods. Similar results pattern was also observed in 5-FU. However, microscopic studies on their cellular morphology proved otherwise, suggesting that F5 fraction and 5-FU inhibited MCF-7 cells through different pathways. Tamoxifen-treated cells, on the other hand, were able to show induction of cell apoptosis and DNA ladder formation. Fortunately, antiproliferation assay showed that F5 fraction was able to stop normal cell proliferation as well as VEGF-induced cell proliferation of HUVECs. These aforementioned results verified that the F5 fraction of* C. amabile* leaf CE possesses a cytostatic ability through antiproliferation activity rather than induction of cell apoptosis.

## Figures and Tables

**Table 1:** Ratio used in the preparation of different solvent mixtures in fractionation of chloroform extract (CE). The volume prepared for each solvent mixture was 200mL and each ratio was prepared in triplicate.

	Percentage (%)										
Petroleum ether	100	80	60	40	20	0	0	0	0	0	0
Chloroform	0	20	40	60	80	100	80	60	40	20	0
Methanol	0	0	0	0	0	0	20	40	60	80	100

Fraction	Wax	F1	F1	F2	F2	F3	F3	F4	F4	F5	F6

**Table 2:** IC_50_ values of different extracts and different fractions of leaves *Crinum amabile*, against various cancer cell lines, using MTS assay. All extract and fraction were exposed for 48 hours with the highest concentration tested being 200 *μ*g/mL. All results were presented as mean ± SEM, of three individual experiments. Triplicate samples were tested in each individual experiment (n = 9).

Samples	IC_50_, (*μ*g/mL)				
	Breast		Colon		Leukemia
	MCF-7	MDA-MB-231	HCT-116	HT-29	REH
PE	> 200	> 200	> 200	> 200	> 200
CE	116.66±7.99	169.92±6.12	158.69±8.48	126.39±3.96	> 200
ME	> 200	> 200	> 200	> 200	> 200
WE	> 200	> 200	> 200	> 200	> 200

F1 Fraction	> 200	> 200	60.21±0.88	> 200	>200
F2 Fraction	> 200	147.52±2.41	> 200	> 200	117.64±1.50
F3 Fraction	> 200	120.59±1.20	38.49±1.17	> 200	> 200
F4 Fraction	> 200	84.62±3.51	71.08±2.96	> 200	89.88±0.58
F5 Fraction	25.61±3.15	27.15±1.21	40.14±2.53	39.53±1.65	52.69±8.10
F6 Fraction	122.03±5.21	159.93±5.81	> 200	> 200	> 200

5-FU	2.18±0.20	185.58±6.14	2.33±0.07	-	3.63±0.10

## Data Availability

The data used to support the findings of this study are available from the corresponding author upon request.
